# 2-[(*Z*)-(3-{[(*Z*)-2-Hy­droxy-3,5-diiodo­benzyl­idene]amino}­propyl­imino)­meth­yl]-4,6-diiodo­phenol

**DOI:** 10.1107/S160053681203214X

**Published:** 2012-07-18

**Authors:** Hadi Kargar, Reza Kia, Amir Adabi Ardakani, Muhammad Nawaz Tahir

**Affiliations:** a Department of Chemistry, Payame Noor University, PO Box 19395-3697 Tehran, I. R. of IRAN; b Department of Chemistry, Science and Research Branch, Islamic Azad University, Tehran, Iran; c Ardakan Branch, Islamic Azad University, Ardakan, Iran; d Department of Physics, University of Sargodha, Punjab, Pakistan

## Abstract

In the title compound, C_17_H_14_I_4_N_2_O_2_, there are two intra­molecular O—H⋯N hydrogen bonds, which make *S*(6) ring motifs. In the crystal, there are no significant inter­molecular inter­actions present.

## Related literature

For standard bond lengths, see: Allen *et al.* (1987 ▶). For hydrogen-bond motifs, see: Bernstein *et al.* (1995 ▶). For background to Schiff base ligands, see, for example: Kargar *et al.* (2011 ▶); Kia *et al.* (2010 ▶). For a related structure, see: Kargar *et al.* (2012 ▶).
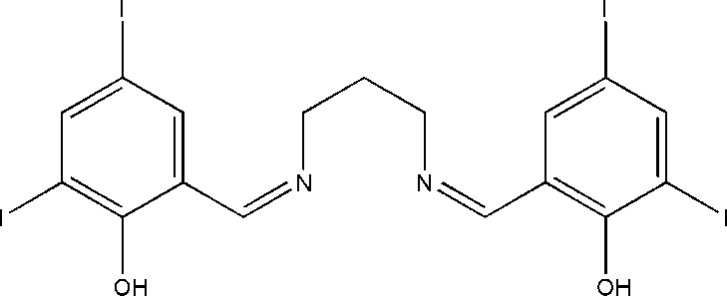


## Experimental

### 

#### Crystal data

C_17_H_14_I_4_N_2_O_2_
*M* *_r_* = 785.90
Monoclinic, 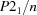

*a* = 4.5578 (3) Å
*b* = 16.5095 (11) Å
*c* = 27.2417 (18) Å
β = 91.736 (4)°
*V* = 2048.9 (2) Å^3^
*Z* = 4
Mo *K*α radiation
μ = 6.10 mm^−1^
*T* = 291 K
0.30 × 0.14 × 0.14 mm

#### Data collection

Bruker SMART APEXII CCD area-detector diffractometer
Absorption correction: multi-scan (*SADABS*; Bruker, 2005 ▶) *T*_min_ = 0.262, *T*_max_ = 0.482
16049 measured reflections
4458 independent reflections
2832 reflections with *I* > 2σ(*I*)
*R*_int_ = 0.047

#### Refinement

*R*[*F* ^2^ > 2σ(*F* ^2^)] = 0.035
*wR*(*F* ^2^) = 0.059
*S* = 0.98
4458 reflections
226 parameters
H-atom parameters constrained
Δρ_max_ = 0.61 e Å^−3^
Δρ_min_ = −0.72 e Å^−3^

### 

Data collection: *APEX2* (Bruker, 2005 ▶); cell refinement: *SAINT* (Bruker, 2005 ▶); data reduction: *SAINT*; program(s) used to solve structure: *SHELXS97* (Sheldrick, 2008 ▶); program(s) used to refine structure: *SHELXL97* (Sheldrick, 2008 ▶); molecular graphics: *SHELXTL* (Sheldrick, 2008 ▶); software used to prepare material for publication: *SHELXTL* and *PLATON* (Spek, 2009 ▶).

## Supplementary Material

Crystal structure: contains datablock(s) global, I. DOI: 10.1107/S160053681203214X/su2471sup1.cif

Structure factors: contains datablock(s) I. DOI: 10.1107/S160053681203214X/su2471Isup2.hkl

Supplementary material file. DOI: 10.1107/S160053681203214X/su2471Isup3.cml

Additional supplementary materials:  crystallographic information; 3D view; checkCIF report

## Figures and Tables

**Table 1:** Hydrogen-bond geometry (Å, °)

*D*—H⋯*A*	*D*—H	H⋯*A*	*D*⋯*A*	*D*—H⋯*A*
O1—H1⋯N1	0.90	1.76	2.569 (5)	148
O2—H2⋯N2	0.89	1.75	2.564 (5)	150

**Fig. 1.**
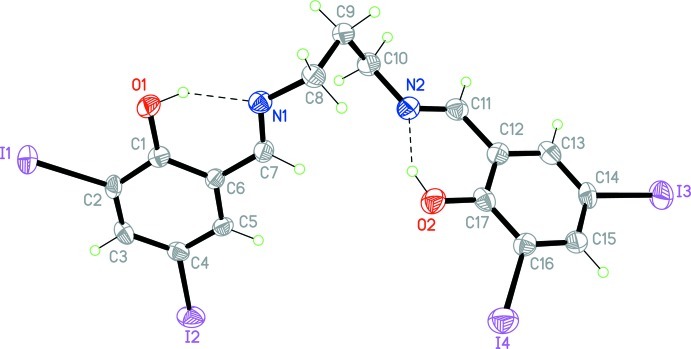
The molecular structure of the title compound, showing 40% probability displacement ellipsoids and the atomic numbering.

## supplementary crystallographic information

### Comment

In continuation of our work on the crystal structures of Schiff base ligands (Kargar *et al.*, 2011; Kia *et al.*, 2010), we synthesizes and carried out the X-ray structure analysis of the title compound.

In the title compound, Fig. 1, a potential tetradentate Schiff base ligand, there are two intramolecular O—H···N hydrogen bonds (Table 1) that make *S(6)* ring motifs (Bernstein *et al.*, 1995). The bond lengths (Allen *et al.*, 1987) and angles are within the normal ranges and are comparable to those reported for a similar structure (Kargar *et al.*, 2012).

In the crystal, there are no significant intermolecular interactions present.

### Experimental

The title compound was synthesized by adding 3,5-dibromosalicylaldehyde (2 mmol) to a solution of propylenediamine (1 mmol) in ethanol (30 ml). The mixture was refluxed with stirring for 30 min. The resultant solution was filtered. Light-yellow prismatic single crystals of the title compound, suitable for *X*-ray structure determination, were obtained by recrystallization from ethanol by slow evaporation of the solvents at room temperature over several days.

### Refinement

The OH H atoms were located in a difference Fourier map and were constrained to ride on the parent O atom with U_iso_(H) = 1.5U_eq_(O). The C-bound H atoms were included in calculated positions and treated as riding atoms: C-H = 0.93 and 0.97 Å, with U_iso_(H) = 1.2U_eq_(C).

### Crystal data

**Table:**

C_17_H_14_I_4_N_2_O_2_	*F*(000) = 1432
*M**_r_* = 785.90	*D*_x_ = 2.548 Mg m^−^^3^
Monoclinic, *P*2_1_/*n*	Mo *K*α radiation, λ = 0.71073 Å
Hall symbol: -P 2yn	Cell parameters from 2453 reflections
*a* = 4.5578 (3) Å	θ = 2.5–27.5°
*b* = 16.5095 (11) Å	µ = 6.10 mm^−^^1^
*c* = 27.2417 (18) Å	*T* = 291 K
β = 91.736 (4)°	Needle, light-yellow
*V* = 2048.9 (2) Å^3^	0.30 × 0.14 × 0.14 mm
*Z* = 4	

### Data collection

**Table:**

Bruker SMART APEXII CCD area-detector diffractometer	4458 independent reflections
Radiation source: fine-focus sealed tube	2832 reflections with *I* > 2σ(*I*)
Graphite monochromator	*R*_int_ = 0.047
φ and ω scans	θ_max_ = 27.1°, θ_min_ = 1.4°
Absorption correction: multi-scan (*SADABS*; Bruker, 2005)	*h* = −5→5
*T*_min_ = 0.262, *T*_max_ = 0.482	*k* = −21→17
16049 measured reflections	*l* = −34→34

### Refinement

**Table:**

Refinement on *F*^2^	Primary atom site location: structure-invariant direct methods
Least-squares matrix: full	Secondary atom site location: difference Fourier map
*R*[*F*^2^ > 2σ(*F*^2^)] = 0.035	Hydrogen site location: inferred from neighbouring sites
*wR*(*F*^2^) = 0.059	H-atom parameters constrained
*S* = 0.98	*w* = 1/[σ^2^(*F*_o_^2^) + (0.0161*P*)^2^] where *P* = (*F*_o_^2^ + 2*F*_c_^2^)/3
4458 reflections	(Δ/σ)_max_ = 0.002
226 parameters	Δρ_max_ = 0.61 e Å^−^^3^
0 restraints	Δρ_min_ = −0.72 e Å^−^^3^

### Special details

**Table:**

Geometry. All esds (except the esd in the dihedral angle between two l.s. planes) are estimated using the full covariance matrix. The cell esds are taken into account individually in the estimation of esds in distances, angles and torsion angles; correlations between esds in cell parameters are only used when they are defined by crystal symmetry. An approximate (isotropic) treatment of cell esds is used for estimating esds involving l.s. planes.
Refinement. Refinement of F^2^ against ALL reflections. The weighted R-factor wR and goodness of fit S are based on F^2^, conventional R-factors R are based on F, with F set to zero for negative F^2^. The threshold expression of F^2^ > 2sigma(F^2^) is used only for calculating R-factors(gt) etc. and is not relevant to the choice of reflections for refinement. R-factors based on F^2^ are statistically about twice as large as those based on F, and R- factors based on ALL data will be even larger.

### Fractional atomic coordinates and isotropic or equivalent isotropic displacement parameters (Å^2^)

**Table:**

	*x*	*y*	*z*	*U*_iso_*/*U*_eq_	
I1	−0.84612 (9)	1.18172 (2)	0.147565 (15)	0.05342 (13)	
I2	−0.80900 (9)	0.88529 (2)	0.274765 (14)	0.05293 (13)	
I3	0.66705 (10)	0.36716 (2)	0.091322 (16)	0.06029 (14)	
I4	0.86332 (9)	0.67131 (2)	0.208721 (14)	0.05504 (13)	
O1	−0.4461 (7)	1.0651 (2)	0.08517 (12)	0.0406 (9)	
H1	−0.3177	1.0345	0.0688	0.061*	
O2	0.4376 (7)	0.7341 (2)	0.12415 (12)	0.0446 (9)	
H2	0.2946	0.7497	0.1030	0.067*	
N1	−0.1123 (8)	0.9443 (2)	0.06648 (15)	0.0350 (10)	
N2	0.0681 (9)	0.7303 (3)	0.05131 (15)	0.0395 (11)	
C1	−0.5088 (10)	1.0271 (3)	0.12709 (18)	0.0303 (12)	
C2	−0.6977 (10)	1.0640 (3)	0.16025 (19)	0.0322 (12)	
C3	−0.7811 (11)	1.0236 (3)	0.20139 (19)	0.0377 (13)	
H3	−0.9105	1.0480	0.2226	0.045*	
C4	−0.6736 (11)	0.9465 (3)	0.21164 (17)	0.0341 (13)	
C5	−0.4784 (10)	0.9102 (3)	0.18129 (18)	0.0329 (12)	
H5	−0.4028	0.8593	0.1890	0.040*	
C6	−0.3933 (10)	0.9501 (3)	0.13875 (18)	0.0294 (12)	
C7	−0.1787 (10)	0.9141 (3)	0.10729 (18)	0.0342 (13)	
H7	−0.0857	0.8666	0.1175	0.041*	
C8	0.1172 (11)	0.9065 (3)	0.03753 (18)	0.0378 (13)	
H8A	0.2494	0.8767	0.0594	0.045*	
H8B	0.2299	0.9487	0.0220	0.045*	
C9	−0.0043 (11)	0.8493 (3)	−0.00185 (18)	0.0391 (14)	
H9A	0.1530	0.8340	−0.0232	0.047*	
H9B	−0.1519	0.8776	−0.0217	0.047*	
C10	−0.1411 (11)	0.7724 (3)	0.01916 (18)	0.0415 (14)	
H10A	−0.3131	0.7867	0.0374	0.050*	
H10B	−0.2030	0.7368	−0.0075	0.050*	
C11	0.1244 (10)	0.6563 (3)	0.04552 (19)	0.0375 (13)	
H11	0.0295	0.6283	0.0200	0.045*	
C12	0.3351 (10)	0.6128 (3)	0.07772 (19)	0.0351 (13)	
C13	0.3936 (11)	0.5311 (3)	0.07004 (19)	0.0407 (14)	
H13	0.3015	0.5036	0.0441	0.049*	
C14	0.5879 (11)	0.4914 (3)	0.1010 (2)	0.0387 (13)	
C15	0.7236 (11)	0.5313 (3)	0.14057 (19)	0.0381 (13)	
H15	0.8510	0.5032	0.1618	0.046*	
C16	0.6701 (11)	0.6115 (3)	0.14830 (18)	0.0352 (13)	
C17	0.4795 (11)	0.6551 (3)	0.11703 (19)	0.0347 (13)	

### Atomic displacement parameters (Å^2^)

**Table:**

	*U*^11^	*U*^22^	*U*^33^	*U*^12^	*U*^13^	*U*^23^
I1	0.0713 (3)	0.0303 (2)	0.0585 (3)	0.0101 (2)	−0.0011 (2)	−0.0040 (2)
I2	0.0675 (3)	0.0569 (3)	0.0348 (2)	−0.0083 (2)	0.00783 (19)	0.0078 (2)
I3	0.0820 (3)	0.0365 (2)	0.0627 (3)	0.0121 (2)	0.0063 (2)	−0.0041 (2)
I4	0.0633 (3)	0.0545 (3)	0.0467 (2)	−0.0014 (2)	−0.0092 (2)	−0.0021 (2)
O1	0.051 (2)	0.033 (2)	0.038 (2)	0.0030 (17)	0.0072 (18)	0.0107 (18)
O2	0.057 (2)	0.031 (2)	0.046 (2)	0.0008 (18)	−0.0050 (19)	0.0028 (19)
N1	0.034 (3)	0.037 (3)	0.034 (3)	−0.001 (2)	0.004 (2)	0.003 (2)
N2	0.042 (3)	0.038 (3)	0.039 (3)	0.001 (2)	0.001 (2)	0.002 (2)
C1	0.034 (3)	0.023 (3)	0.033 (3)	−0.010 (2)	−0.004 (3)	0.001 (3)
C2	0.036 (3)	0.019 (3)	0.042 (3)	0.001 (2)	−0.003 (3)	−0.004 (3)
C3	0.044 (3)	0.032 (3)	0.038 (3)	−0.001 (3)	0.006 (3)	−0.010 (3)
C4	0.041 (3)	0.033 (3)	0.028 (3)	−0.006 (3)	0.001 (3)	−0.002 (3)
C5	0.038 (3)	0.031 (3)	0.029 (3)	0.001 (2)	−0.008 (3)	0.002 (3)
C6	0.032 (3)	0.023 (3)	0.032 (3)	−0.001 (2)	−0.007 (2)	−0.002 (2)
C7	0.035 (3)	0.031 (3)	0.037 (3)	0.005 (2)	−0.004 (3)	−0.001 (3)
C8	0.037 (3)	0.042 (3)	0.035 (3)	0.002 (3)	0.007 (3)	0.002 (3)
C9	0.041 (3)	0.046 (4)	0.031 (3)	0.011 (3)	0.001 (3)	0.001 (3)
C10	0.038 (3)	0.046 (4)	0.039 (3)	0.004 (3)	−0.007 (3)	−0.005 (3)
C11	0.034 (3)	0.044 (4)	0.035 (3)	−0.001 (3)	0.004 (3)	0.000 (3)
C12	0.033 (3)	0.036 (3)	0.037 (3)	−0.001 (3)	0.007 (3)	0.007 (3)
C13	0.046 (4)	0.039 (3)	0.038 (3)	−0.002 (3)	0.010 (3)	−0.005 (3)
C14	0.041 (3)	0.026 (3)	0.049 (4)	0.004 (2)	0.011 (3)	−0.003 (3)
C15	0.043 (3)	0.037 (3)	0.035 (3)	0.004 (3)	0.005 (3)	0.003 (3)
C16	0.038 (3)	0.031 (3)	0.037 (3)	−0.002 (3)	0.003 (3)	0.006 (3)
C17	0.039 (3)	0.030 (3)	0.035 (3)	−0.004 (3)	0.014 (3)	0.000 (3)

### Geometric parameters (Å, º)

**Table:**

I1—C2	2.083 (5)	C6—C7	1.447 (6)
I2—C4	2.103 (5)	C7—H7	0.9300
I3—C14	2.100 (5)	C8—C9	1.521 (6)
I4—C16	2.091 (5)	C8—H8A	0.9700
O1—C1	1.341 (5)	C8—H8B	0.9700
O1—H1	0.9004	C9—C10	1.533 (6)
O2—C17	1.334 (5)	C9—H9A	0.9700
O2—H2	0.8944	C9—H9B	0.9700
N1—C7	1.264 (6)	C10—H10A	0.9700
N1—C8	1.468 (6)	C10—H10B	0.9700
N2—C11	1.260 (6)	C11—C12	1.468 (7)
N2—C10	1.452 (6)	C11—H11	0.9300
C1—C2	1.405 (6)	C12—C13	1.392 (6)
C1—C6	1.409 (6)	C12—C17	1.422 (7)
C2—C3	1.367 (6)	C13—C14	1.372 (7)
C3—C4	1.390 (7)	C13—H13	0.9300
C3—H3	0.9300	C14—C15	1.392 (7)
C4—C5	1.370 (6)	C15—C16	1.364 (6)
C5—C6	1.397 (6)	C15—H15	0.9300
C5—H5	0.9300	C16—C17	1.397 (7)
			
C1—O1—H1	108.6	C8—C9—H9A	108.9
C17—O2—H2	107.0	C10—C9—H9A	108.9
C7—N1—C8	119.9 (4)	C8—C9—H9B	108.9
C11—N2—C10	121.4 (4)	C10—C9—H9B	108.9
O1—C1—C2	119.7 (4)	H9A—C9—H9B	107.7
O1—C1—C6	121.7 (5)	N2—C10—C9	110.7 (4)
C2—C1—C6	118.5 (5)	N2—C10—H10A	109.5
C3—C2—C1	120.4 (4)	C9—C10—H10A	109.5
C3—C2—I1	119.6 (4)	N2—C10—H10B	109.5
C1—C2—I1	120.0 (4)	C9—C10—H10B	109.5
C2—C3—C4	120.4 (5)	H10A—C10—H10B	108.1
C2—C3—H3	119.8	N2—C11—C12	122.1 (5)
C4—C3—H3	119.8	N2—C11—H11	118.9
C5—C4—C3	120.7 (5)	C12—C11—H11	118.9
C5—C4—I2	119.7 (4)	C13—C12—C17	120.1 (5)
C3—C4—I2	119.6 (4)	C13—C12—C11	120.6 (5)
C4—C5—C6	119.8 (5)	C17—C12—C11	119.3 (5)
C4—C5—H5	120.1	C14—C13—C12	119.6 (5)
C6—C5—H5	120.1	C14—C13—H13	120.2
C5—C6—C1	120.0 (5)	C12—C13—H13	120.2
C5—C6—C7	120.5 (4)	C13—C14—C15	120.9 (5)
C1—C6—C7	119.4 (5)	C13—C14—I3	120.0 (4)
N1—C7—C6	122.9 (5)	C15—C14—I3	119.1 (4)
N1—C7—H7	118.6	C16—C15—C14	120.1 (5)
C6—C7—H7	118.6	C16—C15—H15	119.9
N1—C8—C9	113.1 (4)	C14—C15—H15	119.9
N1—C8—H8A	109.0	C15—C16—C17	121.1 (5)
C9—C8—H8A	109.0	C15—C16—I4	120.5 (4)
N1—C8—H8B	109.0	C17—C16—I4	118.4 (4)
C9—C8—H8B	109.0	O2—C17—C16	120.3 (5)
H8A—C8—H8B	107.8	O2—C17—C12	121.6 (5)
C8—C9—C10	113.3 (4)	C16—C17—C12	118.1 (5)
			
O1—C1—C2—C3	175.1 (4)	C11—N2—C10—C9	−127.5 (5)
C6—C1—C2—C3	−3.9 (7)	C8—C9—C10—N2	−55.5 (6)
O1—C1—C2—I1	−6.0 (6)	C10—N2—C11—C12	−179.9 (4)
C6—C1—C2—I1	174.9 (3)	N2—C11—C12—C13	−179.5 (5)
C1—C2—C3—C4	1.8 (7)	N2—C11—C12—C17	0.5 (8)
I1—C2—C3—C4	−177.0 (4)	C17—C12—C13—C14	0.9 (8)
C2—C3—C4—C5	1.2 (7)	C11—C12—C13—C14	−179.0 (5)
C2—C3—C4—I2	−179.4 (3)	C12—C13—C14—C15	1.1 (8)
C3—C4—C5—C6	−2.0 (7)	C12—C13—C14—I3	177.9 (4)
I2—C4—C5—C6	178.6 (3)	C13—C14—C15—C16	−1.6 (8)
C4—C5—C6—C1	−0.2 (7)	I3—C14—C15—C16	−178.4 (4)
C4—C5—C6—C7	177.6 (4)	C14—C15—C16—C17	0.0 (8)
O1—C1—C6—C5	−175.9 (4)	C14—C15—C16—I4	178.2 (4)
C2—C1—C6—C5	3.1 (7)	C15—C16—C17—O2	−177.7 (5)
O1—C1—C6—C7	6.3 (7)	I4—C16—C17—O2	4.1 (6)
C2—C1—C6—C7	−174.7 (4)	C15—C16—C17—C12	2.0 (7)
C8—N1—C7—C6	177.2 (4)	I4—C16—C17—C12	−176.3 (3)
C5—C6—C7—N1	174.0 (4)	C13—C12—C17—O2	177.2 (5)
C1—C6—C7—N1	−8.2 (7)	C11—C12—C17—O2	−2.9 (7)
C7—N1—C8—C9	96.3 (5)	C13—C12—C17—C16	−2.5 (7)
N1—C8—C9—C10	−68.6 (5)	C11—C12—C17—C16	177.5 (4)

### Hydrogen-bond geometry (Å, º)

**Table:**

*D*—H···*A*	*D*—H	H···*A*	*D*···*A*	*D*—H···*A*
O1—H1···N1	0.90	1.76	2.569 (5)	148
O2—H2···N2	0.89	1.75	2.564 (5)	150
